# Nanocrystalline materials: recent advances in crystallographic characterization techniques

**DOI:** 10.1107/S2052252514020818

**Published:** 2014-10-28

**Authors:** Emilie Ringe

**Affiliations:** a Department of Materials Science and NanoEngineering, Rice University, 6100 Main Street MS325, Houston, TX 77005, USA

**Keywords:** nanocrystalline materials, plasmonics, shape prediction models

## Abstract

Most properties of nanocrystalline materials are shape-dependent, providing their exquisite tunability in optical, mechanical, electronic and catalytic properties. An example of the former is localized surface plasmon resonance (LSPR), the coherent oscillation of conduction electrons in metals that can be excited by the electric field of light; this resonance frequency is highly dependent on both the size and shape of a nanocrystal. An example of the latter is the marked difference in catalytic activity observed for different Pd nanoparticles. Such examples highlight the importance of particle shape in nanocrystalline materials and their practical applications. However, one may ask ‘how are nanoshapes created?’, ‘how does the shape relate to the atomic packing and crystallography of the material?’, ‘how can we control and characterize the external shape and crystal structure of such small nanocrystals?’. This feature article aims to give the reader an overview of important techniques, concepts and recent advances related to these questions. Nucleation, growth and how seed crystallography influences the final synthesis product are discussed, followed by shape prediction models based on seed crystallography and thermodynamic or kinetic parameters. The crystallographic implications of epitaxy and orientation in multilayered, core-shell nanoparticles are overviewed, and, finally, the development and implications of novel, spatially resolved analysis tools are discussed.

## Abstract 2

This feature article reviews the control and understanding of nanoparticle shape from their crystallography and growth. Particular emphasis is placed on systems relevant for plasmonics and catalysis.

## 1. Introduction   

Nanocrystalline materials abound in nature and technology, bridging the gap between molecular sized and macroscale objects. Nanocrystals are typically defined as anything with crystal domains in the 1–1000 nm range, including small grain polycrystalline substances, nanofabricated surfaces, polymer micelles and nanoparticles; each of them has varied applications, from drug delivery, to supercapacitors, sensors and catalysts. A variety of crystallographic characterization techniques can be applied to these materials, albeit their unique length scales, well above what is typically studied with traditional diffraction techniques, renders them challenging to probe. Several new developments, both in synthesis and characterization, have recently emerged, and in this feature article, we will highlight the crystallography of small, synthesized nanocrystals and review how electron-based techniques can be used to characterize nanoparticles relevant for catalysis and plasmonic applications.

While exciting characterization advances appear regularly, the idea of using nanoparticles for catalysis and plasmonics is age-old. The Lycurgus cup, medieval stained glass and Faraday’s ruby gold fluid are striking examples of how artists and scientists have utilized the optical properties of gold and silver nanostructures. Of course, very little was known about their size, shape and crystallography until the advent of electron microscopy. Similarly, the ability to image nanoscaled objects shed light on the existence and importance of extremely small particles in efficient catalysts. From the late twentieth century, scientists have realised and explored the many facets of nanoscale phenomena, propelled by and encouraging the development of new crystallographic and imaging tools.

Most properties of nanocrystalline materials are shape-dependent, providing their exquisite tunability in optical, mechanical, electronic and catalytic properties (Osawa & Kawata, 2001 ▶; Bell, 2003 ▶; Wiley *et al.*, 2006 ▶). An example of the former is localized surface plasmon resonance (LSPR; Liebsch, 1993 ▶; Osawa & Kawata, 2001 ▶; Haes & Van Duyne 2004 ▶; Ozbay, 2006 ▶), the coherent oscillation of conduction electrons in metals that can be excited by the electric field of light. This oscillation gives rise to enhanced fields at the surface of the particle, which forms the basis of surface-enhanced spectroscopies (Fort & Grésillon, 2008 ▶; Stiles *et al.*, 2008 ▶; Brown *et al.*, 2013 ▶); its frequency is also dependent on the surrounding medium, providing a truly nanoscale sensing opportunity (Mayer & Hafner, 2011 ▶). The LSPR resonance frequency is not only acutely dependent on the size and composition of the material, but also on its shape (Kelly *et al.*, 2003 ▶). Such factors also influence, in different systems, the catalytic activity of nanoparticles, as has been shown in, for example, the electron-transfer reaction between hexacyanoferrate (III) ions and thiosulfate ions on platinum particles. In this case, the activation energy for tetrahedral nanoparticles is nearly half the activation energy for cubic nanoparticles (Narayanan & El-Sayed, 2004 ▶). Shape and size also strongly influence the bandgap of nanosized direct bandgap semiconductors, from the well known CdS, CdSe and CdTe to the more recent core-shell architectures (Brus, 1983 ▶; Reiss *et al.*, 2009 ▶; Doane & Burda, 2012 ▶; Shirasaki *et al.*, 2013 ▶). Such examples highlight the importance of particle shape in nanocrystalline materials and their practical applications, where specific properties can be enabled and optimized by tuning the particle shape.

Despite its overwhelming importance, the control and understanding of nanocrystalline shape remains an active area of research with much yet to be learned: we are still asking ourselves ‘how are nanoshapes created?’, ‘how does the shape relate to the atomic packing and crystallography of the material?’, ‘how can we control and characterize the external shape and crystal structure of such small nanocrystals?’. Through this feature article, we will provide the most current answers to these questions and give the reader an overview of important techniques and recent advances in the field, focusing on electron microscopy and diffraction characterization of nanocrystalline materials. The first part discusses nucleation, growth and how seed crystallography influences the final synthesis product and how shape can be predicted from seed crystallography and thermodynamic or kinetic parameters. Then, the crystallographic implications of epitaxy and orientation in multilayered, core-shell nanoparticles are overviewed. Finally, new advanced techniques and their potential applications are reviewed, followed by an overview of future possibilities.

## 2. Crystallography of nanoparticle growth and shape   

The final shape of nanoparticles is related to a variety of growth conditions and thermodynamic factors, providing a rich yet complex system. Prominent and convenient ways to obtain shape-controlled nanocrystalline materials include fabrication, ‘top down’ approaches (lithography, evaporation *etc.* ) and synthesis, ‘bottom-up’ approaches (solution or gas-based reduction of metal precursors). The former typically generates polycrystalline shapes with poorly controlled grain orientation and irreproducible crystallography, such that it will not be discussed here. The latter has produced a vast body of knowledge in the past 20 years, and is now a not completely but well understood tool. Bottom-up approaches indeed now allow the reproducible synthesis of a collection of shapes and architectures, including pure metals, alloys, core-shell and anisotropic nanocrystals.

To understand the shape of nanoparticles and how to attain them, we must indeed understand their growth, from a single atom to the final, stable product. In order for a particle to grow, it must first nucleate; this process can be described by the Pound and La Mer nucleation model (Pound & La Mer, 1952 ▶). In a solution containing a metal salt (providing *M* ^*n*+^) and a reducing agent, metal ions are reduced and the concentration of *M* ^0^ rises until a point of critical limiting supersaturation, and any further increase in concentration will lead to the onset of nucleation of small metal clusters. Rapid self-nucleation then occurs, lowering abruptly the *M* ^0^ concentration until a slower, diffusion-limited, growth regime is reached and continues until the metal supplies are exhausted. It is believed that at small sizes, below a few nm, the atomic positions within the particle fluctuate, sampling various orientations and shapes. As small particles grow, atomic positions become locked in, and further growth must accommodate the symmetry and crystallography of the nucleus (Ajayan & Marks, 1988 ▶; Dundurs *et al.*, 1988 ▶; Ajayan & Marks, 1989 ▶, 1990 ▶). Thus, shape control starts right at the nucleation stage: single-crystal nuclei will produce single-crystal particles, fivefold twinned seeds will endow their symmetry to the final product, may it be a Marks decahedron, star decahedron, decagonal rod or pentagonal bipyramid (Marks, 1983 ▶; Wiley *et al.*, 2006 ▶; Ringe *et al.*, 2013 ▶). Analogously, particles grown from singly twinned crystals will have at least mirror symmetry. Such particles can simply grow from the initial seeds in the same reaction, or the seeds can be transferred to a different, perhaps less vigorous, medium for further growth without nucleation. This process has been very successful in providing shape control, as it effectively decouples the selection of nucleus crystallography from the selection of preferentially expressed crystallographic facets.

**Figure 1:**
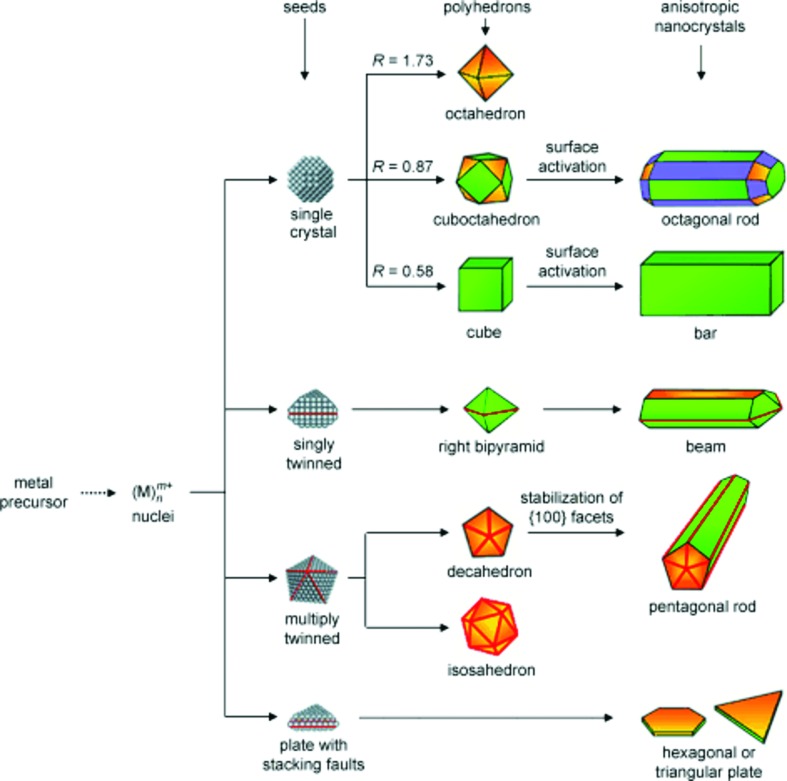
Schematic illustrating the various families of shapes accessible to f.c.c. materials. The initial reduction and nucleation forms a seed that is either single crystalline, singly twinned, multiply twinned or planar with multiple parallel staking faults (which often fall under the umbrella of ‘singly twinned’). Green, purple and orange represent {100}, {110} and {111} surfaces, and the parameter *R* is the ratio between the growth velocity of {100} and {111} facets. Adapted from Xia *et al.* (2009 ▶).

**Figure 2:**
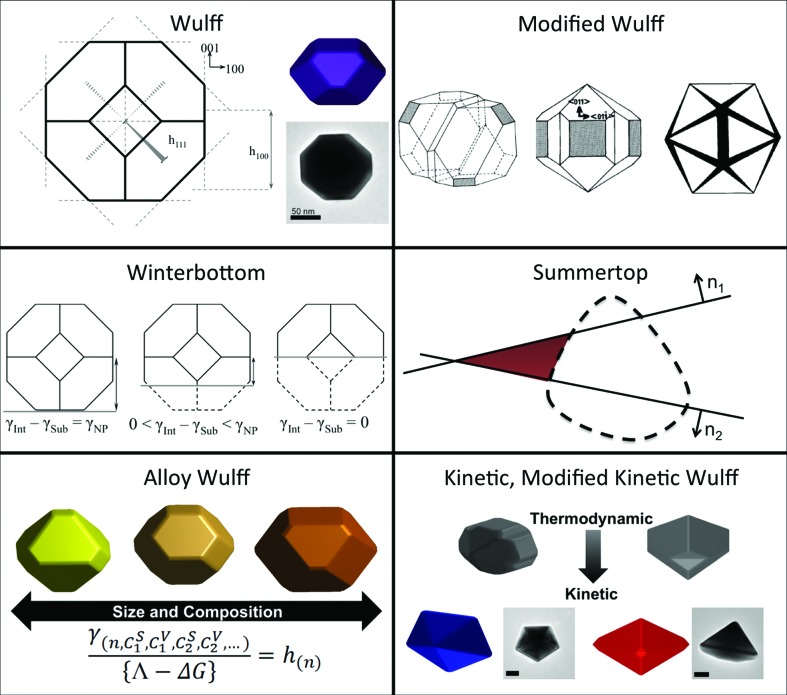
Wulff construction and related models. Adapted and reprinted with permission from Marks (1983 ▶), Enterkin *et al.* (2011 ▶ ) and Ringe *et al.* (2011 ▶, 2013 ▶). Copyright (2011, 2013) American Chemical Society.

Conveniently, many metals and simple alloys crystallize in a face-centered cubic (f.c.c.) phase, thus sharing similar twinning patterns promoted by low energy {111} twin boundaries. This similitude causes common nucleation and growth patterns, leading to a shared family of possible shapes, as shown in Fig. 1 ▶. Given a seed crystallography, may it be single crystal, single twin, twin with multiple parallel staking faults, fivefold twin or twenty-fold twin, a variety of shapes can be obtained. Such shapes can be rationalized using one of the variants of the Wulff construction, an energy-minimizing model predicting shape by relating the surface free energy of various crystallographic faces to their relative expression in a particle (lower energy faces are expressed more than higher energy ones) (Wulff, 1901 ▶; von Laue, 1943 ▶; Dinghas, 1944 ▶; Winterbottom, 1967 ▶; Marks, 1983 ▶; Zia *et al.*, 1988 ▶; Ringe *et al.*, 2011 ▶, 2013 ▶). This collection of models, shown in Fig. 2 ▶, directly relates the seed crystallography and the growth conditions to the final shape; experimental growth observations confirm this relationship well. The basic Wulff construction, published over a century ago, is still widely used since nanocrystals with a single crystallographic domain are often encountered in syntheses. A variety of shapes, including octahedra, cuboctahedra (as shown in Fig. 2 ▶), and cubes can thus be obtained depending on the relative surface energy of the crystallographic facets growing from a single-crystal seed (Sun & Xia, 2002 ▶; Wiley *et al.*, 2006 ▶; Skrabalak *et al.*, 2007 ▶; Xiong *et al.*, 2007 ▶; Xia *et al.*, 2009 ▶). Given the tendency of many crystals, especially f.c.c., to form low-energy twin boundaries, multiply twinned nanoparticles are rather common. The (thermodynamic) modified Wulff construction (Marks, 1983 ▶, 1984 ▶) or the recently derived kinetic modified Wulff construction (Ringe *et al.*, 2013 ▶) (Fig. 2 ▶) can, in the appropriate cases, be used to predict and understand the shape of twinned f.c.c. crystals. Combined, they can generate the full spectrum of twinned shapes, from the thermodynamic Marks decahedron and star decahedron to the kinetic decagonal rods, pentagonal bipyramids and thin triangular platelets (Wiley *et al.*, 2006 ▶; Zhang *et al.*, 2009 ▶; Ringe *et al.*, 2012 ▶). Another extension of the Wulff construction is the introduction of a substrate, either as a flat interface as in the Winterbottom construction (Winterbottom, 1967 ▶) or surrounding the particle as in the summertop (crystal in a corner) construction (Zia *et al.*, 1988 ▶). These models are important, as when particles are supported when grown, the difference between the interfacial free energy γ­_Int_ (defined as the difference between the sum of the facet and substrate free energy and the binding energy between the two, γ_nanoparticle facet_ + γ_substrate_ − γ_bond_) and substrate free energy energy γ_substrate_ can cause dramatic changes in shapes, as observed for Pt particles grown on {100} faces of strontium titanate (Enterkin *et al.*, 2011 ▶). In Pt/SrTiO_3_ case, there is a significant affinity between the nanoparticle and the substrate, leading them to form a modified Wulff shape ‘sinking in’ the substrate. Finally, a more recent model has been developed to consider the unique effects of segregation in single-crystal nanoalloys (Ringe *et al.*, 2011 ▶). In such systems, if the particles are small enough, atoms segregating at the surface effectively starve the bulk and change the bulk free energy, creating a non-negligible contribution to the total energy. Given the size-dependence of the ratio of atoms on the surface and the bulk, different sizes have different equilibrium surface/bulk compositions, hence different shapes (Ringe *et al.*, 2011 ▶). The effects are not simply limited to the shape of the particle but also affect the energy and surface composition, such that a full treatment of segregation and bulk composition changes should be included when performing calculations related to nanoscale alloys; this should even apply to small-grain bulk samples, where segregation at the grain boundaries occur. There is a limited body of supporting evidence for the alloy effects; more targeted experiments aiming at obtaining accurate shape and surface information are needed to better understand small segregating alloys. Yet overall, Wulff constructions have been extremely successful at explaining particle shape because of their simplicity and flexibility, and their profound grounding in nanoparticle twinning and crystallography.

## 3. Crystallinity in nanoparticles   

While some of the theories discussed in the above section remain to be confirmed, experimental results abound for pure metal synthesis. While most, if not all, results can be explained through seed crystallography and different growth velocity or surface energies, ultimately we are interested in rational control of the shape of the final product and its stability with respect to shape changes. Seed twinning control is the very first step in this process, and given the limited understanding of the early stages of nucleation, design of seed synthesis remains rare. Various observations, however, highlight the importance of the surfactant used during initial nucleation. For instance, gold seeds are predominantly single crystals or multiply twinned (fivefold) when chloroauric acid (HAuCl_4_) is reduced with sodium borohydride (NaBH_4_) in the presence of either cetyl trimethylammonium bromide (CTAB) or sodium citrate (NaH_2_C_6_H_5_O_7_), respectively (Jana *et al.*, 2001 ▶; Nikoobakht & El-Sayed, 2003 ▶; Liu & Guyot-Sionnest, 2005 ▶). Another handle on the crystallography of the final product is seed selection. Indeed, when performing a two-step growth (seeds first, then transfer into a growth medium), it is possible to choose seeds by selectively etching them with small amounts of acids, as reported by the Xia group (Lu *et al.*, 2009 ▶; Xia *et al.*, 2009 ▶). Twinned seeds etch faster than single-crystal ones, likely due to their different chemical potential, such that in the right conditions one obtains almost pure single-crystal seeds from an initially heterogeneous product.

Once the seed crystallography is selected, surface energy and kinetic conditions determine which shape amongst the family of related shapes will be primarily obtained, as per the Wulff construction or its variants described above. For a single-crystal seed of f.c.c. material, for example, the Wulff construction predicts that an octahedron will be obtained if {111} surfaces are more stable, while a cube will be obtained in the case of greater {100} stability. To control which crystallographic faces are preferentially expressed and deliberately modify the balance of surface expression (thus shape) the relative surface energy or growth velocity must be manipulated. This is typically done in solution by the use of surfactants, which inhibit the growth of specific facets due to their selective adsorption. For example, it was shown that polyvinylpyrrolidone (PVP) selectively binds {100} facets, while citrate binds the {111} facets of silver nanoparticles. Thus, cubes are routinely obtained for PVP-based syntheses. Underpotential deposition (UPD), the tendency for a small amount of material (typically sub-monolayer) to deposit on a substrate at a potential less negative than the equilibrium potential, can also slow or stop the growth of specific crystallographic faces. This approach is particularly well suited to Au synthesis, as Ag can deposit on low-energy faces. With this technique the synthesis of concave Au cubes with remarkably high-index faces ({720}-type) was possible (Zhang *et al.*, 2010 ▶), as well as a number of other shapes such as {117}-bound Au pentagonal bipyramids (Liu & Guyot-Sionnest, 2005 ▶). In complex syntheses, involving two or more growth steps, the shape of the seed in the final step is usually well defined, *e.g.* a cube or a rod; curvature effects then become important in directing the growth. Less steric hindrance from the adsorbed surfactants can promote growth around high curvature areas, as is observed in overgrown Au rods (Song *et al.*, 2005 ▶).

**Figure 3:**
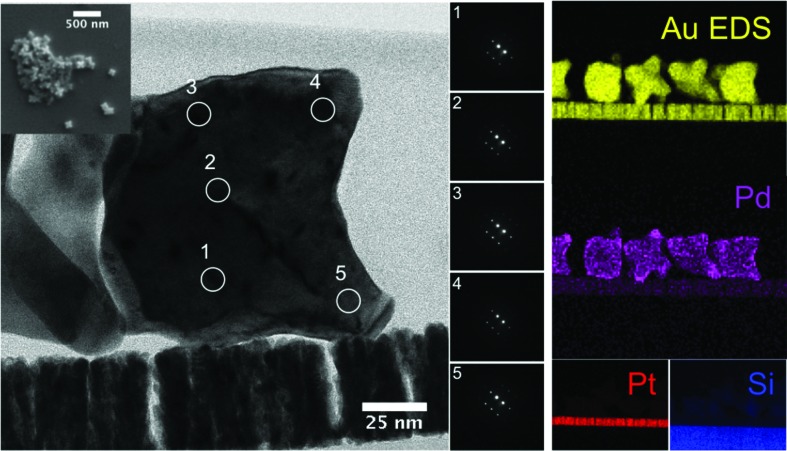
Au/Pd octopods obtained by seed-mediated, co-reduction synthesis. Left inset: overview of the nanoparticles. Left: thin lamellae of particle deposited on a coated silicon chip, prepared with a focused ion beam (Hitachi NB5000) and imaged with a scanning transmission electron microscope (Hitachi HD-2700). Center: diffraction mapping of the lamellae, showing the particle to be a single crystal; lattice imaging also confirms this observation. Right: energy dispersive X-ray spectroscopy maps; note the high Pd concentration at the tips of the particles.

Seed crystallography defines which shapes are accessible in one-component systems but do not always apply to the crystal structure of multicomponent, layered nanocrystals. Cases in which the substrate (core) and film (shell) are epitaxically grown can be found for materials with very similar lattice parameters. For example, crystallographically interesting AuPd octopods, which have excellent, simultaneous catalysis and plasmonic properties, have been recently synthesized through a multi-step reaction (DeSantis *et al.*, 2012 ▶; DeSantis & Skrabalak, 2012 ▶). In this reaction, small (< 10 nm) gold seeds are first obtained, and then grown in a different medium into < 50 nm cubes, cuboctahedra or octahedra. The third and final step involves co-reduction of Au and Pd onto the large cores. Given the excellent miscibility of Au and Pd, an alloy is formed at the surface of the core and may even penetrate it. This alloy is, as far as we observed, in perfect epitaxy with the core. Electron diffraction mapping with a nm-sized probe on a focused-ion beam thinned nanoparticle confirms that all the branches and the core are aligned exactly along the same axis, *i.e.* the entire particle is a single crystal (Fig. 3 ▶). Scanning transmission electron microscopy energy dispersive X-ray spectroscopy (STEM-EDS) confirmed, as it has before (DeSantis & Skrabalak, 2012 ▶; DeSantis *et al.*, 2012 ▶) the presence of both Au and Pd on the outer shell of the particle. Diffraction maps of particles ‘as-is’, *i.e.* non-thinned, were also acquired and show identical results, as does lattice imaging at the tips of the particles. Smooth interfaces can also be obtained for AgAu and other fully miscible system capable of forming alloys as well as core-shell nanocrystals (Liu & Guyot-Sionnest, 2004 ▶; Hu *et al.*, 2007 ▶; Seo *et al.*, 2008 ▶; Liu *et al.*, 2009 ▶; Wang *et al.*, 2009 ▶).

**Figure 4:**
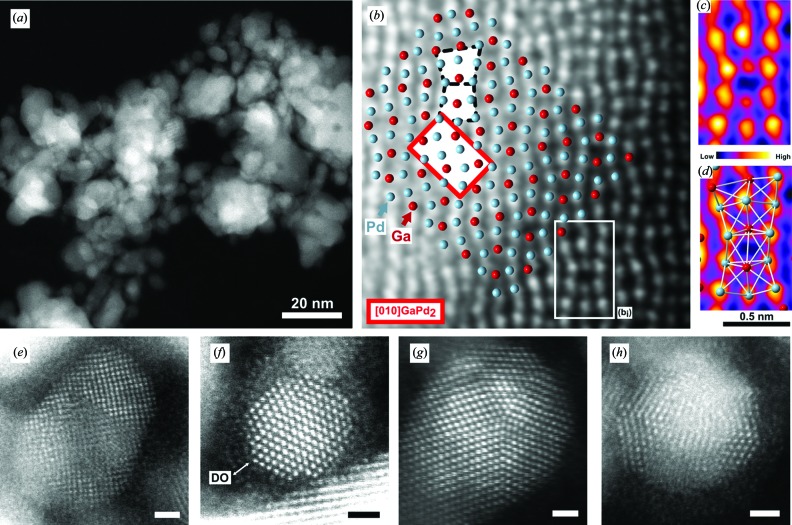
Ga–Pd nanocatalysts revealed by annular dark-field (ADF) imaging in aberration-corrected STEM. (*a*) Low magnification ADF image of the Ga–Pd nanoparticles. (*b*)–(*d*) GaPd_2_ along the [010] zone axis. (*b*) Atomically resolved ADF image (low-pass filtered) on which the structure of GaPd_2_ in the [010] projection is overlaid. The orthorhombic unit cell is outlined in red, and two ‘f.c.c.-like’ units by dashed black lines. (*c*) A selected region (*b_i_*) from (*b*) over which the thickness of the nanoparticle is sufficiently constant to enable a relative comparison of the intensities of the atomic columns in the image. A portion of the GaPd_2_ structure in [010] projection is overlaid in (*d*), confirming that the lower and higher intensity atomic columns in the image correspond to those in the [010] projection of GaPd_2_ that exclusively contain Ga or Pd respectively. (*e*)–(*h*) Small Ga–d nanoparticle morphologies (scale bars: 1 nm). (*e*) Evidence of ‘distorted-f.c.c.-like’ nanocrystalline structure characteristic of Ga–Pd in the intermetallic state. (*f*) Twinned truncated octahedral nanoparticle exhibiting pseudo-{111}_f.c.c._ re-entrant facets and surface defects. An oxidic disordered over-layer (DO) decorating the surface of the nanoparticle is indicated. (*g*) Decahedral and (*h*) icosahedral fivefold twinned morphologies viewed down their five- and twofold symmetry axes, respectively. Reproduced with permission from Leary *et al.* (2013 ▶).

Most of the materials discussed thus far have single composition or random alloy f.c.c. structures, and thus similar accessible shapes. Such materials have interesting applications in catalysis (Pt, Pd) and optical devices (Au, Ag, Cu, Al). Another interesting material for catalysis, more specifically selective hydrogenation, has recently emerged: intermetallic Ga–Pd compounds (Armbrüster *et al.*, 2010 ▶). In foundational studies, use of bulk or powdered model systems led to insights into the relationship between structural and electronic properties and catalytic performance. Through high-resolution imaging, spectroscopic and three-dimensional tomographic TEM studies, significant insight into the crystallography of unsupported Ga–Pd nanocatalysts, GaPd_2_ in particular, has been possible (Leary *et al.*, 2012 ▶, 2013 ▶). Especially the directly interpretable and chemically sensitive imaging achievable using annular dark-field imaging in aberration-corrected STEM has proven invaluable (Leary *et al.*, 2012 ▶, 2013 ▶). Further to direct verification of the distinctive intermetallic structure in nano-sized particles (Fig. 4 ▶), catalytically significant and crystallographically intriguing deviations compared to the ‘ideal’ bulk crystal have been revealed. These include strong surface segregation, lattice relaxation and particularly interesting morphologies of the small (< 10 nm) particles that comprise both nanocrystalline ‘f.c.c.-like’ and fivefold twinned structures (Fig. 4 ▶). Particularly interesting is that GaPd_2_ can be interpreted in terms of a distorted and chemically ordered ‘f.c.c.-like’ structure, and consideration of the observed fivefold twinning in that context presents an intriguing crystallographic puzzle. Such twinning and segregation in well defined alloys will certainly prompt further theoretical work; the quality of the data obtained appears to be sufficient to challenge and verify new modeling approaches.

**Figure 5:**
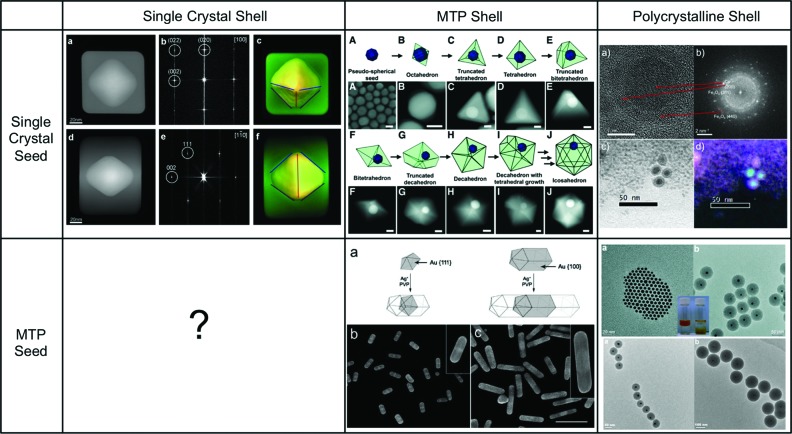
Possible core-shell crystallography and examples. Au core–Ag shell nanostructures (top left), reproduced with permission from Gómez-Graña *et al.* (2013 ▶), (top middle) reproduced with permission from Langille *et al.* (2012 ▶), (bottom middle) reprinted with permission from Seo *et al.* (2008 ▶), Co core Fe_3_O_4_ shell top right, adapted from Knappett *et al.* (2013 ▶) with permission of The Royal Society of Chemistry, and silica-coated Au cores (bottom left, adapted with permission from Han *et al.*, 2008 ▶). Copyright (2013, 2008) American Chemical Society. Copyright (2012) AAAS.

Catalytic nanoparticles, such as GaPd, need not be single phase. In fact multifunctional designed systems can often more easily be obtained with core-shell architectures. In our work and those of others, a magnetic or plasmonic core can be coated with a catalytically active material to obtain either magnetically separable particles, or to couple plasmonic sensing or enhancement with heterogeneous catalysis (Lee *et al.*, 2006 ▶; Tsuji *et al.*, 2006 ▶; DeSantis & Skrabalak, 2012 ▶; Knappett *et al.*, 2013 ▶). In core-shell syntheses, the crystallinity of the core and shell are typically independent. Single crystals can evolve to single-crystal core-shell particles, as discussed above for AuPd structures and as shown in Fig. 5 ▶ for Au–Ag core-shell nanoparticles. Single crystal cores can also be coated by polycrystalline shells, as in the case of silica-coated Au or Fe_3_O_4_-coated Co (Liz-Marzán *et al.*, 1996 ▶; Obare *et al.*, 2001 ▶; Mine *et al.*, 2003 ▶; Knappett *et al.*, 2013 ▶); well defined multiply twinned particles can also hold similarly multiply twinned shell (Seo *et al.*, 2008 ▶) or a polycrystalline coating (Liz-Marzán *et al.*, 1996 ▶; Obare *et al.*, 2001 ▶; Mine *et al.*, 2003 ▶; Knappett *et al.*, 2013 ▶). Polycrystalline coatings tend to be conformal such that a coated cube will be a slightly rounded cube, and coating a sphere will result in a sphere. Once deposited on a substrate, it is also possible to coat nanoparticles with, for example, atomic layer deposition (ALD), which also provides a conformal, polycrystalline shell.

Due to epitaxy effects, it appears difficult to produce single-crystal shells on twinned cores, however, two approaches may solve, more or less satisfactorily, this puzzle. First, it is possible to grow a shell that apparently has a single crystalline shape from twinned seeds. The Kitaev group has indeed synthesized cubic geometries from the growth of silver on twinned silver platelets, albeit these are arguably twinned cubes (McEachran & Kitaev, 2008 ▶). A potential approach to coating twinned seeds with single crystalline shells would be to bypass epitaxy and orientation effects *via* a buffer layer between the core and shell, such as the amorphous carbon layer observed in Co core Fe_3_O_4_ shell nanostructures (Knappett *et al.*, 2013 ▶). While single-crystal shells on twinned seeds might be challenging, the reverse has recently been observed. Indeed, single-crystal seeds can lead to the formation of well defined multiply twinned shells. Langille and co-workers grew a layer of Ag on single crystalline Au seeds in the presence of a {111}-directing surfactant, bis-(*p*-sulfonatophenyl)-phenylphosphine dihydrate potassium salt (BSPP), and obtained decahedral and icosahedral motifs, as shown in Fig. 5 ▶ (Langille *et al.*, 2012 ▶). While they did not directly perform diffraction studies, the Fourier transform of high-resolution images showing lattice fringes indicate that the seeds are indeed crystalline and fully embedded in the Ag shell. However, it is difficult to tell exactly where the seed is within the nanoparticles, which would provide further information about the growth anisotropy. Such valuable insight could be provided by studies using electron tomography, yielding three-dimensional information akin that obtained for Au core–Ag shell particles also shown in Fig. 5 ▶.

Crystallography in nanoparticles is overall very important to control the final shape; however, in core-shell particles many strategies have been developed to either bypass or utilize the underlying crystallography. Particle synthesis and crystallography control have seen an exciting decade, and will likely continue to grow and become an increasingly well understood science. Specifically, *in-situ* studies of nucleation and growth are likely to provide further handles on seed control, while material design and growth through the assembly of single layer materials will allow new structures and shapes to be reproducibly obtained.

## 4. Advanced shape and composition characterization techniques for nanocrystalline materials   

‘Seeing is believing’. For nanostructures, this represents a difficult challenge, as most common techniques do not allow localized characterization; the advent of electron microscopy and microanalysis, however, has promoted rapid changes in the depth and breadth of our understanding of nanoscale systems. In this section, we will review some of the most exciting advancements in X-ray based techniques and discuss several new electron characterization techniques; for a full review the reader is encouraged to consult some of the recent work and reviews in the field (Billinge & Levin, 2007 ▶; Hohenester *et al.*, 2009 ▶; García de Abajo, 2010 ▶; Thomas & Midgley, 2010 ▶; Egerton, 2011 ▶).

X-rays are a mainstay in crystallography, and remain so at the nanoscale, but not without limitations. For example, X-ray powder diffraction can be a very useful albeit ensemble-averaged technique in which peak broadening can be converted to information about grain size. Most times, however, the broadening of soft X-rays by small-grain materials blurs the information content already diminished by the low diffracted intensity. However, several new X-ray techniques have emerged to address such limitations. Coherent X-ray diffraction imaging (CXDI) (Miao *et al.*, 1999 ▶; Chapman & Nugent, 2010 ▶) can, when coupled with the extremely high intensity achieved by X-ray free electron lasers, provide information at the single large (> 100 nm) particle level. Using this technique, the size of Ag nanocubes and Ag/Au nanoboxes supported on a silicon nitride membrane was determined (Takahashi *et al.*, 2013 ▶). This technique, akin to the diffract-before-destroy approach, is currently limited to very bright photon sources, but it may become a powerful tool to study nanostructured and polycrystalline materials in the future. A particular opportunity in this case is the intrinsic ability to probe the size of the grains as a function of the Bragg angle, potentially revealing preferential orientations in nanocrystalline materials. The pulses emitted by X-ray free-electron lasers are not only very intense and coherent, but also very short (500 to < 10 fs) (Emma *et al.*, 2010 ▶), such that time-resolved experiments on single particles are becoming a reality. For instance, coherent acoustic phonons (lattice vibrations) in gold nanoparticles have been imaged in three dimensions using the shifts in the Bragg peak associated with lattice expansion and contraction (Clark *et al.*, 2013 ▶). Again, these measurements are on large (300–400 nm) nanoparticles, but improvements and optimization will lead to the ability to probe smaller and smaller particles, as was observed in optical absorption and scattering spectroscopies of small metal nanoparticles, for which current detection limits are a few nanometers (Ringe *et al.*, 2013 ▶).

**Figure 6:**
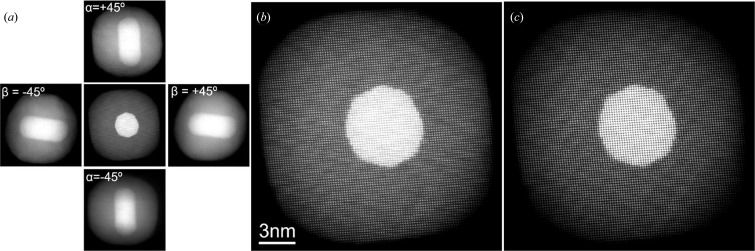
High-resolution HAADF-STEM image of Au rod/Ag shell nanoparticle. (*a*) Five projection images from tilts along orthogonal angles; (*b*) detailed view of the zero tilt projections; (*c*) refined model of the zero tilt image. Reprinted with permission from Goris *et al.* (2013 ▶). Copyright (2013) American Chemical Society.

While the shape of single nanostructures can be well understood from the two-dimensional projection provided by an electron micrograph, some structures are inherently more difficult to understand. Non-platonic solids make interpreting images somewhat challenging, and this can be rendered even more difficult in the presence of stacking/layering geometries such as core-shell or aggregate structures. To surmount this obstacle, three-dimensional reconstructions based on electron microscopy images have proven invaluable (Midgley & Dunin-Borkowski, 2009 ▶). In electron tomography, images of a complex object are acquired at small intervals of rotation about a tilt axis, and they are used to reconstruct the shape of the particle. Care must be taken, however, to choose a signal that satisfies the projection requirement; for crystalline materials, electron tomography is typically carried out with STEM-ADF rather than bright field TEM, since the latter introduces artifacts caused by diffraction contrast and Fresnel fringes. Using such techniques, the distribution of Ru/Pt bimetallic nanocatalysts on nanoporous silica support was characterized, revealing preferential locations of the particles within the support (Ward *et al.*, 2007 ▶). Indeed, particles within the interior of the support appear to be more likely to be located on saddle-shaped sites, while particles on the outside of the support preferred cup-like areas. These findings have profound implications on our understanding of catalysts, as the substrate and substrate interactions play a crucial role, sometimes believed to be as important as size and shape, in the catalytic activity and selectivity. As previously discussed, electron tomography has also been useful in the investigation of plasmonic materials, as shown in Fig. 5 ▶ for a Au core–Ag shell nanostructure. In this case, the reconstruction unambiguously shows the location of the seed within the particle. Recently, much higher resolution tomograms have been obtained, reaching atomic resolution (Goris *et al.*, 2012 ▶; Scott *et al.*, 2012 ▶; Van Dyck *et al.*, 2012 ▶; Goris *et al.*, 2013 ▶) with HAADF-STEM. For example, the crystallographic defects within Pt nanoparticles (Chen *et al.*, 2013 ▶) were observed, and the atomic arrangement, crystallographic orientation, and smooth interface of Au rods–Ag shell structures (Fig. 6 ▶; Goris *et al.*, 2013 ▶). As the technique matures, atomic resolution will become more commonplace and more easily implemented to complex systems. However, it remains unclear whether atomic resolution EDS or EELS will ever be a quantitative reality: factors such as channeling, re-channeling, and beam broadening are genuinely hard to avoid and account for, and much more work will need to be done in the field before it becomes established.

Beyond characterizing the shape, location and crystal structure of nanocrystalline materials, electron microscopy-based tomography has recently been utilized to unravel the composition, as well as the electric and magnetic fields in and around nanoscale objects. Indeed, electron holography-based reconstructions have shown the electrostatic potential profile around a *p*–*n* junction (Twitchett-Harrison *et al.*, 2007 ▶), while the reconstruction of electron-energy loss signals around a silver nanocube provided a three-dimensional view of its many plasmon modes (Nicoletti *et al.*, 2013 ▶). We believe that three-dimensional imaging, by itself as well as coupled with other spatially resolved analysis, will play a pivotal role in enhancing our understanding of nanocrystalline materials. The development of new approaches and algorithms, for example the introduction of electron diffraction tomography and related techniques (Kolb *et al.*, 2007 ▶; Boullay *et al.*, 2013 ▶; Mayence *et al.*, 2014 ▶) will enhance the technique’s applicability to nanocrystalline systems; as routine resolution and ease of use advance, tomography is expected to occupy and ever-increasing place in nanoscience.

## 5. Conclusion   

The crystallography of nanoparticles controls, from the very beginning of their formation, their properties, may these be catalytic, plasmonic or electronic, due to the shape control it provides. In multistep syntheses, seeds of chosen crystallography can be used, imparting a choice of final shapes determined by the relative energy or growth velocities of the various facets, as predicted by Wulff constructions. However, in core-shell particles, the twinning pattern of the seed does not necessarily match that of the shell, providing even greater flexibility in the synthesis of multicomponent/multifunctional materials. Such complex materials will become increasingly popular, both for optical and catalysis applications, as will the localized, quantitative approaches needed to characterize them, in particular electron-based approaches and three-dimensional reconstruction techniques.
